# How Fast Does a Signal Propagate through Proteins?

**DOI:** 10.1371/journal.pone.0064746

**Published:** 2013-06-06

**Authors:** Hui T. Young, Scott A. Edwards, Frauke Gräter

**Affiliations:** 1 CAS-MPG Partner Institute and Key Laboratory for Computational Biology, Shanghai, P. R. China; 2 Graduate School of Chinese Academy of Sciences, Beijing, P. R. China; 3 Heidelberg Institutes for Theoretical Studies gGmbH, Heidelberg, Germany; 4 College of Physics and Technology, Shenzhen University, Shenzhen, Guangdong, P. R. China; Consiglio Nazionale delle Ricerche, Italy

## Abstract

As the molecular basis of signal propagation in the cell, proteins are regulated by perturbations, such as mechanical forces or ligand binding. The question arises how fast such a signal propagates through the protein molecular scaffold. As a first step, we have investigated numerically the dynamics of force propagation through a single (Ala)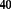 protein following a sudden increase in the stretching forces applied to its end termini. The force propagates along the backbone into the center of the chain on the picosecond scale. Both conformational and tension dynamics are found in good agreement with a coarse-grained theory of force propagation through semiflexible polymers. The speed of force propagation of 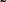
50Å ps^−1^ derived from these simulations is likely to determine an upper speed limit of mechanical signal transfer in allosteric proteins or molecular machines.

## Introduction

Mechanical force has long been recognized as one of the major factors that regulates biological function not only on the macroscopic level such as tissues and organs, but also on the microscopic level such as individual cells and proteins [1]–[3]. Cells and their constitutes are subjected to a constantly changing environment to which they are required to adopt in a dynamic and timely way. The dynamic response of a molecular system like a protein or protein complex to an external force has been extensively studied in experiments, simulations and theory [4]–[8]. While these processes of protein conformational changes or protein unfolding upon force application typically exhibit time scales from microseconds to seconds or even hours [9], the time scale of the force to propagate into the molecular structure is probably several orders of magnitude faster. The velocity of force propagation, i.e. the speed of sound, can be considered as an upper limit for the mechanical response of biomolecules, and thus is of fundamental interest in order to explore the dynamic consequences of external perturbations. One intriguing example is the helical fibers in hair cell bundles of vertebrate inner ear that serve as force transducers and are postulated to give rise to the exquisite frequency sensitivity ranging from 20 to 20 000 Hz and remarkably high dynamic range exceeds 100 dB [10]–[12].

**Figure 1:**
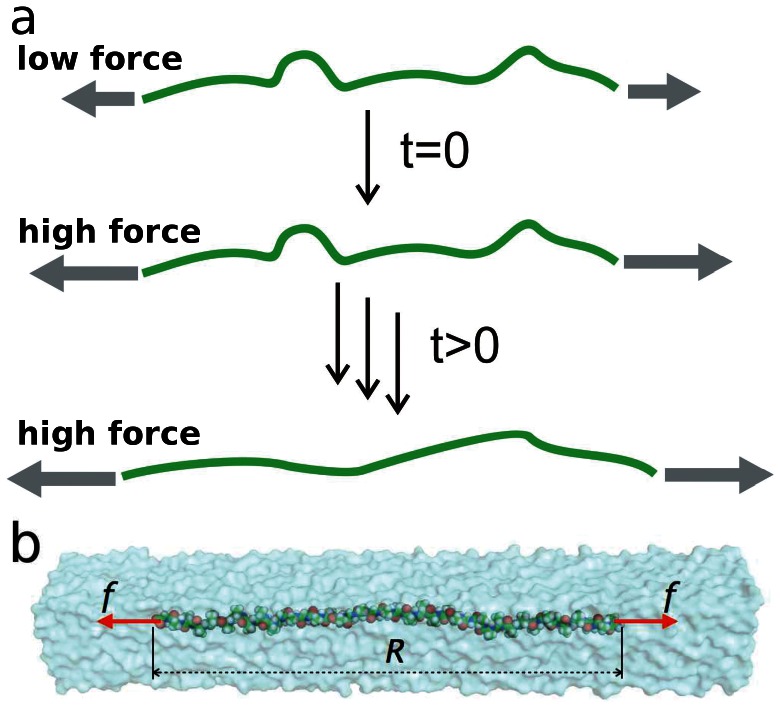
Force propagation along a polypeptide in water. (a) After first equilibrating an (Ala)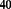 polypeptide under a relatively low stretching force of 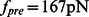 applied to both termini of the polypeptide, we then suddenly increase the stretching force by a factor of 10 to 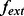. As the polymer is straightened, backbone tension propagates from the two termini into the center of the chain. (b) Structure used in the MD simulation. The (Ala)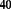 polypeptide is surrounded by explicit solvent molecules. A constant force, 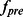 or 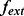 is applied to the terminal C-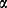 atoms, and the end-to-end distance 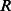 of the peptide is measured.

Unfortunately, force propagation is inherently difficult to measure experimentally. Doing so not only requires excellent time resolution, one also has to either introduce force-sensitive probes into the molecular backbone [13] or resort to indirect and difficult to interpret measures such as force-induced heat dissipation [14]. However, the very same rapidity acting as a roadblock to experimental investigation pushes the subject of force propagation into the realm of atomistic molecular dynamics (MD) simulations, a technique that is limited to very short time scales, but has proven useful for our understanding of protein dynamics [15]. In particular, a good agreement has been observed between experimental and simulated folding rates, suggesting the time scales of non-equilibrium processes investigated by MD simulations to be of a reasonable order of magnitude [16], [17]. We take advantage of this fact by combining atomistic MD simulations of an (Ala)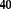 homo-polypeptide under external stretching force with Force Distribution Analysis (FDA) [18], allowing us to track intramolecular forces with perfect temporal and spatial resolution (see Fig. 1).

## Results

### Tension Propagation from MD Simulations

**Figure 2:**
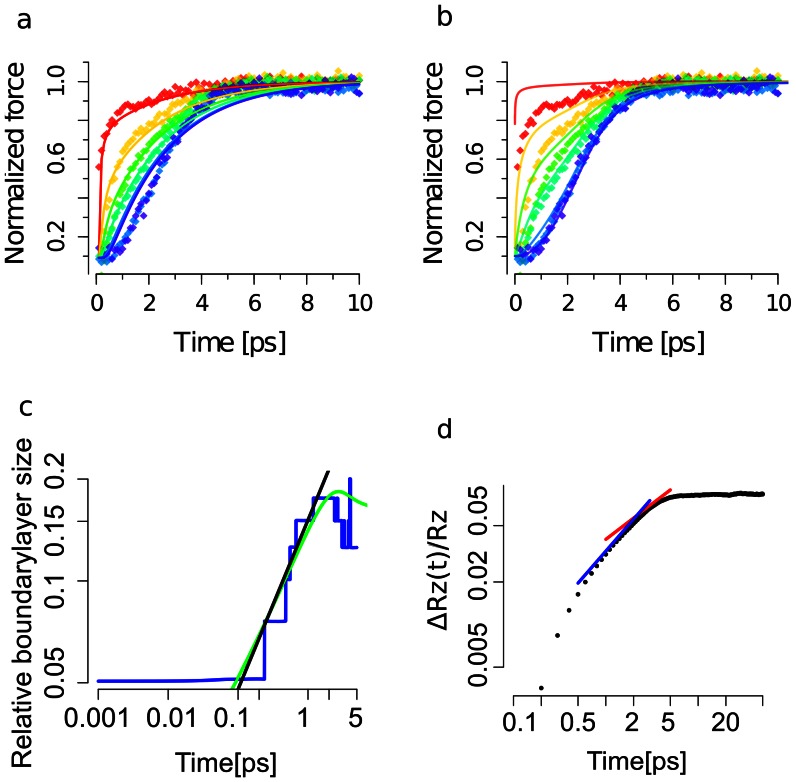
Tension propagation from MD simulations and comparison to dynamic bead-spring and semiflexible chain models. (a) Tension evolution as predicted by a bead-spring model (colored curves) fitted to the tension in Ala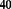 as obtained from MD simulations (colored diamonds). Coloring red, yellow, green yellow, blue and purple show residue pairs 1–2, 4–5, 7–8, 10–11, 15–16 and 20–21, respectively. A manually reduced friction coefficient was used to map the WLC model and numerical results. We note that here forces were averaged over 100 fs time periods for clarity. (b) Tension evolution from the dynamic WLC model (colored curve) fitted to the tension in Ala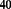 as obtained from MD simulations (colored diamonds). Coloring and averaging as in (a). (c) Boundary layer size 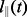 relative to the contour length 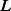 obtained from MD simulations shown in *blue*. Numeric solution to the dynamic WLC model prediction in *green* and 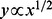 growth law in *black*. (d) Extension 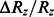 shown as *black dots* compared to two growth laws, 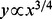 in *blue* and 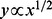 in *red*.

After a sudden increase in the external force applied to the termini of (Ala)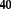 from 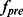 to 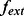, we monitored the tension propagation into the center of the polypeptide by measuring the force between each pair of adjacent residues over time. While the MD simulations at the increased force of 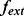 were carried out for 50 ps, we observed the inter-residue forces to equilibrate already within the first 10 ps, and thus restricted the analysis to this time window. As shown in Fig. 2a (diamonds), as expected, the increase in force between pairs of residues is highly non-linear and delayed towards the center of the chain. While the outer residue pairs show a rapid increase in inter-residue force within 1 ps, residue pairs located in the vicinity of the center of the chain exhibit a force increase significantly delayed on the picosecond time scale. A representative dynamic trajectory of (Ala)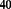 color coded by inter-residue forces is visualized in Movie S1. We next attempted to analyze the numerical results with two distinct polymer models, a bead-spring model and the dynamic Worm-like Chain model.

### Bead-spring Model

**Figure 3:**
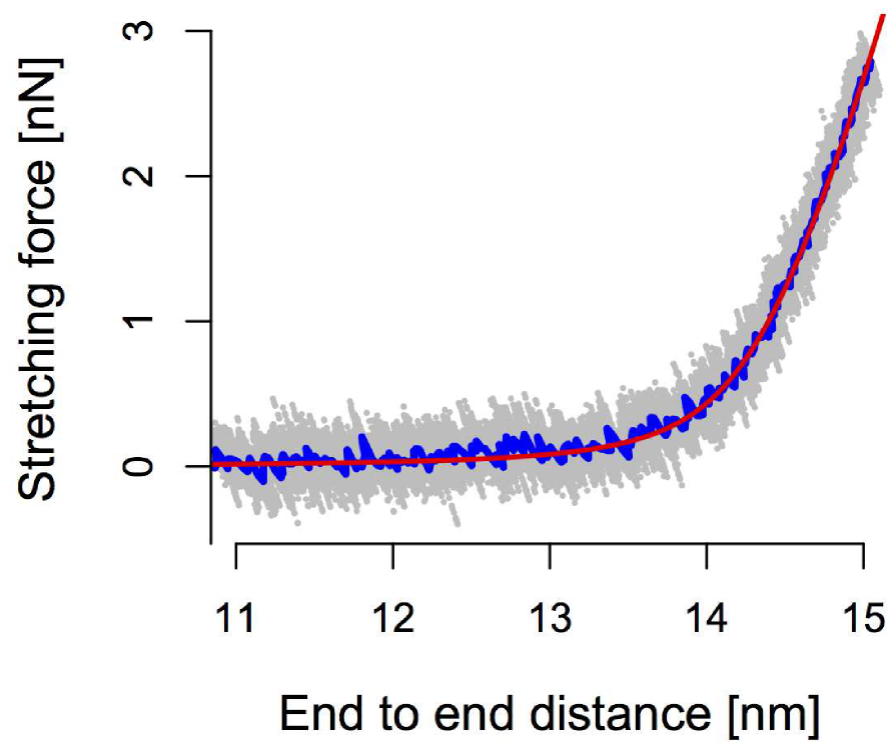
Force-Extension curve of the (Ala)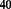 chain (*grey dots*). The average is shown in *blue*, and the fitting result of the worm-like-chain model is shown in *red*. A minimized root mean square residual error of 0.1 nN was obtained by nonlinear least square fitting.

Since protein dynamics are often treated within a linearized bead-spring framework [19], [20], it is natural to assume that tension might propagate diffusively through the protein backbone, in the same way vibrational excitations do [14]. Assuming freely draining hydrodynamics, we solve the corresponding equations of motion.
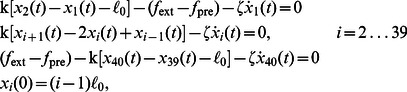
(1)


where 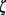 denotes the approximate bead friction coefficient 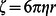, 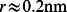 the hydrodynamic radius of a single monomer, 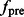 a low force at which the poly-alanine peptide was pre-equilibrated, and 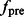 a higher external stretching force. By matching the resulting stretching response 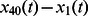 to the end-to-end distance for an intermediate stretching force 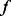, with 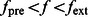, in a corresponding MD force-extension curve (Fig. 3), we determined both the backbone stiffness 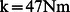 and the contour length per residue 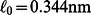. The force-extension profile for (Ala)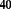 (raw data in grey, averaged data in blue in Fig. 3), obtained from additional MD simulations, gives the stretching force of the chain as a function of its end-to-end distance, and is directly comparable to experimental force-extension data.

Turning now to the time-dependent backbone tension 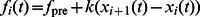, we find that this severely underestimates the actual speed of tension propagation especially for the inner most residues and intermediate time scales (not shown). One might argue that this is due to hydrodynamic cooperativity, as neighboring monomers move in unison, thus possibly shifting their effective friction coefficients towards the infinite-cylinder limit 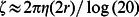 [21], thereby reducing friction by a factor of up to 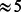. This approximation, however, could only roughly bring our model in line with the MD data (Fig. 2a, Table S1 in File S1). Close to the center, even this manually corrected friction coefficient yields a time-dependent backbone tension that is too high at short times, but too low later on.

The likely reason behind this qualitative discrepancy is the inherently nonlinear stretching behaviour of polymers in thermal equilibrium. If the molecule was perfectly straight, then all the work done by the external stretching force went into pulling the polymer’s atomic constituents further apart, thus acting against powerful chemical binding potentials for which the harmonic approximation holds well beyond the external force levels considered here. As it is, however, Brownian forces always induce a certain amount of contour bending transverse to the polymer main axis, thus effectively shortening it in the longitudinal direction and providing a finite amount of “stored length” that is easily pulled out at forces far below those necessary to stretch the molecular backbone itself. A single stiffness chosen such as to correctly reproduce the overall longitudinal extension will thus always overestimate the actual stiffness, and thus the speed of tension propagation, at low stretching forces, Fig. 3.

### Worm Like Chain (WLC) Model

Previous studies [22] have shown that polypeptides, like many other biopolymers, belong to the class of *semiflexible* polymers, rigid below a certain persistence length 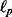 but flexible on long scales 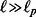. Mathematically, semiflexible polymers are described by the *worm-like chain* model, which yields an accurate expression for the nonlinear force-extension relation [8] (presuming zero backbone extensibility),
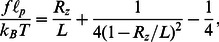
(2)where 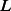 denotes the total polymer length and 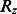 its longitudinal extension. Previous experimental measurements of 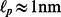 show that we are very close to full extension, 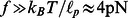, allowing us to simplify the force-extension relation as follows,



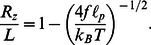
(3)Meanwhile, 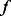 is large enough to stretch the backbone, which we may regard as a linear spring, 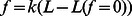, i.e. the full force-extension relation for strong contour straightening and weak backbone stretching reads.
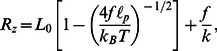
(4)where 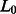 denotes the molecule’s natural contour length, i.e., the longitudinal extension of its energetic ground state. By fitting the above expression to our force-extension data, we obtain 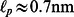, 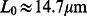 and 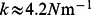. Raising the external force from 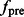 to 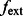 thus stretches the backbone by 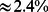, whereas the strain caused by contour straightening is more than twice as large as the harmonic contribution,



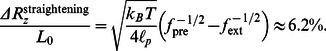
(5)In contrast to the linear bead-spring model, the Wormlike Chain gives rise to very complex short-time behaviour that can only be accounted for by explicit consideration of the nonequilibrium relaxation behaviour of mechanical bending modes [23]–[26]. Fortunately, in the limit of “long” times (in our case anything beyond 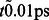) these quickly fluctuating bending modes adapt quasistatically to changes in the backbone tension 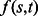, thus allowing us to generalize the above static force-extension relation to spatially and temporally varying tension profiles 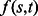,
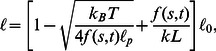
(6)where 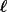 denotes the length of an infinitesimal piece of polymer (of rest length 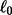).

The corresponding changes in local strain 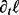 determine the longitudinal velocity gradients 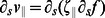 (for further details please refer to [23]–[26]), thus furnishing us with a closed partial differential equation for the time- and space-dependent tension profile,
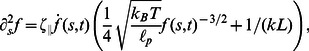
(7)valid for 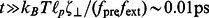 [25] which we solve under the given initial and boundary conditions 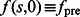, 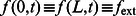. Figure 2b shows the results from the dynamic WLC model for the time-dependent inter-residue forces as obtained from MD simulations (same as Figure 2a). The agreement with our MD data is much improved, especially deep within the chain where forces remain within the regime of nonlinear extensibility for several (Table S1 in File S1).

We also quantified and compared the *boundary layer* as it was defined within the dynamic WLC model in [27]. Following the sudden increase of the external force 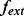, the polymer stretches its contour within a growing boundary layer 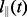. Only within boundary layers, the thermally undulated contour is straightened while in the bulk of the chain tension stays in their original *ground state* defined by 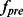. Practically, the boundary layer is defined as the segment of the polymer such that its tension 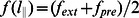. The boundary layer growth in the (Ala)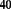 chain relative to its contour length 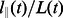 is compared to the model prediction in Figure 2c. We extracted the characteristic parameter 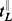 = 1.2 ps, which marks the crossover from the tension *propagation* into the *relaxation* phase. In consistence with the analytical model put forward in [25], for 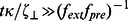, the boundary layer 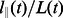 scales as 
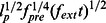
 before two boundary layers from both ends meet each other. Here, 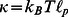 and 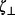 are the bending stiffness and the friction coefficient for transverse motion, respectively. We note that the stair wise appearance of the boundary layer growth in our simulations is due to the discrete nature of the polypeptide chain.

Another observable that we compared to the dynamic WLC model prediction is the change in chain extension 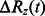. As shown in Figure 2d, its growth is nonlinear and showed a crossover from 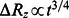 into 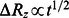, which corresponds to the transition from the tension *propagation* regime into the *relaxation* regime [25].

## Discussion

Through Molecular Dynamics simulations of polyalanine, we have shown that coarse-grained models of semiflexible polymer dynamics yield an accurate description of tension propagation and stretching dynamics even in peptide-sized macromolecules, thus providing a reliable theoretical framework on all relevant length scales.

In a bulk material, the longitudinal signal propagation is given by the Newton-Laplace equation, 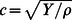, where 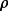 is the density of the material and 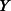 is the Young’s modulus. Our calculations allow to estimate the signal propagation speed for the nanometer-sized single molecule chain of poly-alanine. With a Young’s modulus of 13 GPa at a constant force of 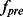 = 166 pN and a 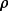 of 507.8 kg m^−3^ derived from van der Waals volume, we obtain a speed of 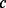 = 51 Å  ps^−1^.

Alternatively, 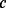 can be estimated from the propagation regime characteristic time scale 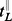 = 1.2 ps in the non-equilibrium MD simulations. Considering that the signal only travels through half the chain length 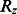, the effective signal propagation speed 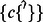 is given by 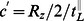 = 56 Å ⋅ ps^−1^. This estimate quantitatively agrees with the estimation from the Newton-Laplace equation, suggesting the theory for macroscopic bulk materials to hold at the level of discrete single molecular chains.

The longitudinal mechanical signal propagation speed we determined here for a stretched peptide is roughly two times of the speed of sound in myoglobin obtained previously [28] and 1.5 times of the value reported for a densely packed 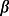-sheet rich proteins in another study [29]. In a stretched peptide, mechanical force almost exclusively transfers through the backbone while in the case of myoglobin or 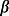-sheet rich proteins it also transfers through a softer network of hydrogen bonds and other non-covalent molecular contacts, which apparently slows down the propagation of forces relative to their transfer through covalent bonds. Mechanical signal propagation plays a pivotal role in protein allostery, which is known to involve much longer time scales from microseconds onwards, but could be envisioned to be partly determined by transmission through either faster backbone or slower non-covalent forces, or a combination thereof. How the picosecond time scale of force propagation through our simplistic single-chain system relates to the dynamics of signal transmission through larger allosteric proteins or molecular machines remains to be resolved.

## Methods

### Molecular Dynamics Simulations

Our simulation encompasses a triclinic box of size 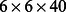 nm with periodic boundary conditions containing besides the (Ala)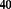 peptide approximately 47000 SPC water molecules [30]. We first perform 2000 energy minimization steps using a steepest descent algorithm, followed by 1 ns of MD simulation to equilibrate the solvent. During solvent equilibration, all protein atoms are held in place by harmonic potentials of stiffness 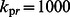 kJ mol^−1^ nm^2^. Next, we remove the artificial constraining potentials and turn on the pre-stretching force, corresponding to linear potentials 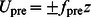 acting on residues 1 and 40, respectively. Another 500ps of MD simulation ensure complete tension equilibration within the peptide. We then increase the external stretching force by a factor of 10 and record all atomic trajectories (with a time resolution of 1fs) during the following 50ps of simulation time.

Using the Force Distribution Analysis (FDA) method implemented based on Gromacs [18], we also record the vectorial forces between each pair of atoms within cut-off distance of each other, again with a time resolution of 1 fs. From this we obtain the sought-after backbone tension by summing all forces between adjacent residues and projecting onto the polymers main axis 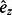.

All simulations are carried out in GROMACS 4.5.4 [31] using the OPLS/AA force field [32] and a 1.0 nm cutoff for non-bonded interactions. Within the 1.0 nm distance, electrostatic interactions are calculated explicitly, while longer ranged electrostatic interactions are evaluated using the Particle Mesh Ewald summation method [33]. Simulations are performed within the NpT ensemble, where temperature is kept constant at 300K by a Nose-Hoover thermostat coupling with a time constant of 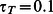 ps [34] and the pressure constraint 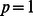 bar is enforced by a Parrinello-Rahman barostat coupling with 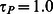 ps and compressibility of 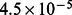 bar^−1^ [35].

For a single nonequilibrium simulation, the magnitude of tension fluctuations measures approximately 6000 pN (Fig. S1a in File S1), thus dwarfing the deterministic average force by a factor of almost 4∶ 1. To arrive at a reasonable signal-to-noise ratio, we average over 100 independent trajectories following the same force-jump protocol, resulting in standard errors of the mean in the range of 20–50 pN (Fig. S1b in File S1), i.e. below the average differences between residue pairs along the chain and in time. We also measure the static force-extension relation by attaching harmonic potentials of stiffness 500 kJ mol^−1^ nm^2^ to the terminal residues and moving them outwards at different constant speeds between 0.5 and 10 nm/ns (constant velocity pulling). The resulting curves are velocity-independent, proving that the pulling velocity is slow enough for backbone tension to equilibrate quasistatically.

## Supporting Information

File S1 **Detailed model fitting parameters.**
(PDF)

Movie S1
**Dynamics of tension propagation after the jump to a high stretching force mapped onto the stretched (Ala)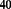 chain.** Elevated force propagates from both ends of the peptide into the central region. The color code from *blue* to *red* indicates lowest to highest inter-residue forces. Only the first 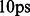 are shown.(MOV)
