# Supplementary material for: How Fast Does a Signal Propagate through Proteins?
Source: PLoS One. 2013 Jun 6;8(6):e64746. doi: 10.1371/journal.pone.0064746 (PMC3675101; doi:10.1371/journal.pone.0064746)
Supplement: File S1 — Detailed model fitting parameters. (PDF) [file pone.0064746.s001.pdf]

Supporting Information File S1

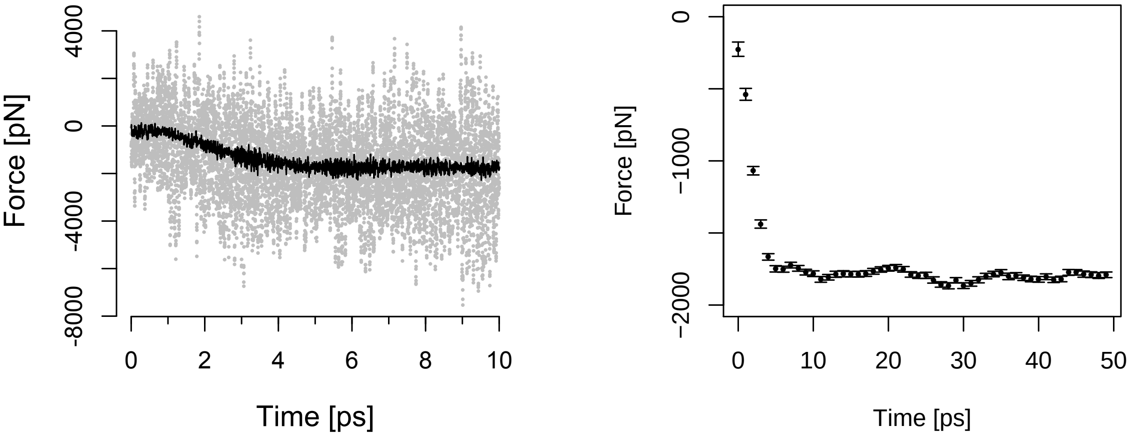

a) b)

Supplementary Figure S1. Force fluctuations and errors. We note that negative forces are attractive. (a) Tension between residues 20 and 21 as a function of time after the sudden jump in the external force. Results from one of the 100 simulations: *grey dots*, average force over all 100 simulations: *solid black*. We note that inter-residue forces are shown for every simulation frame (1 fs) instead of time-averaged forces over every 100 fs as is in Figure 2. (b) Averaged tension between the same residues 20 and 21 during the 50 ps after the force jump. Forces are averaged over 1ps time periods for each trajectory, and then averaged over all 100 trajectories. Error bars show standard errors of the mean.

Supplementary Table S1. Root Mean Squared Error (RMSE) for selected residue pairs obtained from fitting the bead-spring and WLC model to the time-resolved inter-residue forces. The fittings were obtained with the following parameters: For the bead-spring model, a water viscosity of $\eta = 890 \times 10^{-6}$ Pa s and a backbone stiffness of k $=47\,\mathrm{N\,m^{-1}}$ was used; for the WLC model, a persistence length of $\ell_p = 0.7\,\mathrm{nm}$, a contour length of $L_0 = 14.7\,\mathrm{\mu m}$ and a chain elastic stiffness of $k = 4.2\,\mathrm{N\,m^{-1}}$ was used.

Residue pair	1-2	4-5	7-8	10-11	15-16	20-21
Bead-spring	0.052	0.045	0.048	0.042	0.062	0.064
WLC	0.082	0.1	0.078	0.029	0.031	0.031
